# Cost of illness of HER2-positive and metastatic and recurrent HER2-positive breast cancer – a Danish register-based study from 2005 to 2016

**DOI:** 10.1186/s12913-022-08143-7

**Published:** 2022-06-04

**Authors:** M. Spanggaard, J. Olsen, K. F. Jensen, M. Anderson

**Affiliations:** 1 Incentive, Holte Stationsvej 14, 1, 2840 Holte, Denmark; 2 Roche Denmark, Hvidovre, Denmark; 3 Department of Oncology, Copenhagen University Hospital Rigshospitalet, and Danish Breast Cancer Cooperative Group, Copenhagen, Denmark

**Keywords:** Cost of illness, HER2-positive breast cancer, Register analysis, Costs, Nationwide register study

## Abstract

**Background:**

Information and knowledge about cost of illness and labour productivity in patients with HER2-positive early-stage and metastatic breast cancer treated with trastuzumab is limited. The aim of this study was to estimate the direct and indirect costs associated with treatment of HER2-positive breast cancer among patients with early-stage and metastatic breast cancer, treated with trastuzumab, in a 10-year period after diagnosis.

**Materials and methods:**

This study included all Danish HER2-positive breast cancer patients (≥ 18 years) treated with trastuzumab between 2005 and 2016 identified in The Danish Patient Register and the Danish Cancer Register. Furthermore, we identified patients experiencing metastatic or recurrent breast cancer. For the study populations, we estimated total direct costs and indirect costs for one year prior to the breast cancer diagnosis and up to 10 years after diagnosis compared with a group of matched controls free of breast cancer. In addition to The Danish Patient Register and Cancer Register, we applied patient level data from The Civil Registration System, The National Pathology Register, National Health Service Register for Primary Care, Register of Medicinal Product Statistics, Register of Municipal Services, The DREAM database, and Population’s Education Register.

**Results:**

We identified 4,153 HER2-positive breast cancer patients, whereof 27% were identified with metastatic or recurrent breast cancer. During the follow-up period of 10 years, we estimated excess direct costs of EUR 115,000 among the total study population compared to controls; EUR 211,000 among patients with metastases or recurrence; and EUR 89,000 among patients without metastases or recurrence. Direct costs were found to be highest in the first year after diagnosis and also peaked in the year after recurrence. Labour productivity was significantly lower among patients with recurrence 10 years after breast cancer diagnosis compared with controls.

**Conclusions:**

In this study, we estimated the direct and indirect cost associated with HER2-positive breast cancer. The costs were significantly higher during the 10 years after diagnosis compared to the control group, specifically among patients experiencing metastases or recurrence of breast cancer.

**Supplementary Information:**

The online version contains supplementary material available at 10.1186/s12913-022-08143-7.

## Introduction

Breast cancer is the most frequent type of cancer diagnosed among women in European countries, constituting 20–25% of all incident cancer diagnoses [1]. In Denmark, the annual number of cases is approximately 4,700 (age-standardised rate 86.1 per 100,000 women) [2, 3], and worldwide, more than 2 million women were diagnosed with breast cancer in 2018 [4]. The survival rate for breast cancer has increased during the past decade, which is likely attributable to advances in treatments, new surgical techniques, standardisation of treatment protocols, mammography screening and early diagnostics [5]. However, approximately 20% of breast cancer patients experience de novo metastatic breast cancer (metastatic breast cancer at diagnosis) or will experience recurrent cancer, which is usually considered an incurable condition.

About 10–15% of early-stage breast cancer patients have HER2-positive breast cancer [6]. Before the introduction of treatments targeting HER2-positive breast cancer, it was an illness associated with higher rates of disease recurrence and higher mortality than other breast cancer subtypes. However, new targeted therapies have significantly changed the treatment paradigm of patients with early-stage HER2-positive breast cancer [7]. Since 2006, one-year treatment (17 cycles) with trastuzumab in combination with chemotherapy has been offered as standard treatment in Denmark to women with early-stage HER2-positive breast cancer before or after surgery.

Although the various subtypes of breast cancer affect many thousands of women worldwide each year, information and knowledge about the cost of illness and labour productivity of patients with HER2-positive early-stage and metastatic breast cancer treated with trastuzumab is limited. Indirect costs are likely substantial, as a large proportion of HER2-positive cases exit the labour market after treatment [8]. Furthermore, cohort studies comparing HER2-positive cancers with HER2-negative cancers found HER2-positive cases to be more expensive to treat [9], and have higher cost of illness per patient [10].

The previous studies investigating cost of illness for HER2-positive patients have primarily been conducted as cohort studies or through trials. Denmark offers unique options for performing studies on real-world data via access to high-quality, exhaustive register data on the long-term outcomes of implementation of new targeted medicines. Therefore, we used the Danish national registers to investigate the association between HER2-positive breast cancer (with and without metastases or recurrence), extended direct and indirect costs.

## Methods

A retrospective population-based study was designed to analyse direct and indirect costs among Danish women diagnosed with HER2-positive breast cancer in the period 2005 to 2016 who were treated with trastuzumab. Our data included information starting from 2004, in order to observe the change from before diagnosis.

### Data sources

Since 1968, all Danish citizens have been assigned a unique personal identification number, recorded in the Danish Civil Registration System (CRS) [11]. For all individuals, the CRS registers date of birth, gender, vital status, region of residence and family relationships. The CRS enables an identity-secure linkage of information among the Danish national registers. Patient-specific data were collected from the Danish National Patient Register (DNPR), which contains information on all somatic hospitalisations in Denmark since 1977. Moreover, all outpatient activities, emergency room contacts and psychiatric ward contacts, including diagnoses and performed procedures, have been included in the DNPR since 1995 [12]. Information on all tariffs and unit cost estimates for each somatic hospital contact (hospital admissions and outpatient visits) was retrieved from the DNPR, which was available from 2002 onwards. The date of diagnosis of breast cancer was retrieved from the Danish Cancer Register, which contains data on the incidence of cancer and on tumour characteristics among the Danish population, dating back to 1943 [13]. The HER2 status of study participants was obtained from the Danish National Pathology Register, which contains information on all pathology examinations conducted in Denmark since 1997 [14]. Data on primary healthcare services and unit costs were retrieved from the Danish National Health Service Register for Primary Care [15]. Prescription medicine costs were collected from the Register of Medicinal Product Statistics. Acquisition and unit cost estimates were based on the market price, including patient co-payment and public reimbursement. Information regarding home care services was retrieved from the Register of Municipal services. Information of employment was retrieved from the DREAM Database, which is owned by the Danish Ministry of Employment. The DREAM database includes information on weekly labour market public transfer payments, i.e. unemployment benefits or disability payments, for all Danish citizens since 1991. Individuals receiving such a payment are included in the database for the corresponding year, while the remaining members of the workforce are not included.

Information regarding highest obtained education was obtained from the Population’s Education Register [16] and was defined by the highest obtained education at the time of breast cancer diagnosis.

### Institutional setting

The study was conducted in Denmark. Denmark has a tax-financed universal healthcare system, where treatment is free at point of consumption. Hospitals are financed through a combination of block grant transfers and activity-based financing using a DRG-system. Primary care physicians fulfil a gatekeeper-function, where patients need a referral before receiving specialized or hospital care, unless emergency care is necessary. Private health insurance is available, but not widely used. Even if a patient is covered by private health insurance, cancer treatment is provided through the public sector. For prescription drugs, Danish citizens face an annual deductible fee of 2,0.650 Euro. After which, prescription drugs are financed 100% by the state.

All treatment facilities and pharmacies are obligated to report administrative data to the Danish Health Data Authority. The activity data is routinely used for review of hospital activity and reimbursement in accordance with the Danish DRG-system. For cancer treatments, data quality was a stated aim of the Cancer Package II (Implemented 01 January 2006).

### Study population

The study population was defined as women ≥ 18 years diagnosed with incident HER2-positive breast cancer in the period between 2005 and 2016 who were treated with trastuzumab. All women with incident breast cancer were identified in the Danish Cancer Register using the ICD-10 code C50. In the Danish National Pathology Register, information on HER2 status was retrieved using SNOMED code T04 (breast) in combination with either SNOMED code F29603 (HER2 receptor overexpression) or SNOMED code FE13B5 (HER2 gene amplification). In the DNPR, treatment with trastuzumab was identified using treatment code BOHJ13. More than six treatments with trastuzumab in the year following diagnosis resulted in inclusion in the study population.

### Metastatic and recurrent HER2-positive breast cancer

Patients who experienced recurrence or metastatic breast cancer does not qualify as early-stage breast cancer. Metastatic cancer is considered the last stage cancer, while 80% of recurrent cancer cases is identified as metastatic [17]. Hence study participants were assigned to one of two subpopulations: patients who experienced metastatic or recurrent HER2-positive breast cancer within the study period, and patients with early-stage breast cancer who did not experience metastases or recurrence within the study period.

Patients with metastatic HER2-positive breast cancer were defined as patients who were registered with TNM-M code M1 at time of diagnosis or, patients who received more than 17 treatments of trastuzumab in the 13 months after diagnosis [18, 19]. Recurrence was identified in patients who received at least six trastuzumab treatments within the first year after diagnosis, at some point afterward discontinued treatment for at least six months, and then initiated trastuzumab treatment again.

### Control population

To determine the cost of illness, we compare the cost of HER2-positive patients with controls from the Danish population. One control for each patient was randomly selected from the general population via the CRS, matched on age and highest obtained education as of the year of breast cancer diagnosis. All controls were female. The DNPR was used to ensure that all controls were unexposed to breast cancer in the study period. To reduce the risk of introducing bias, we limited the matching criteria to only birth year and highest obtained education. We had in total 91,964 potential controls and the controls were matched exact on a 1:1 ratio based on age and education. If education was obtained abroad or from a not-approved Danish institution, education will be missing in The Education Register. If a patient was observed with missing in this variable, it was matched with a control who also had missing in this variable. Each control was assigned the same index date as the matched case.

### Unit costs and outcome variables

Outcomes for this study included direct and indirect costs attributable to HER2-positive cancer. Direct costs consisted of costs generated from consumption primary care services, hospital care services and home care services. Indirect cost was estimated based on labour market productivity. Additionally, we included prescription medicine as a separate category.

The direct costs were estimated based on services noted in the national registers. For each contact with the healthcare system an observation in the register data is made. This observation contains date and reason for visit, services provided to the patient (including but not limited to consultation, medication/treatment provided in a hospital setting, diagnostic imaging, surgery) and the estimated cost of these services. The cost of all included services are determined by the national tariffs and fees (DRG-tariffs for the hospital sector and fees for the primary healthcare sector).

From the DREAM database yearly employment rates were estimated and subsequently labour productivity values were estimated by multiplying the yearly employment rate, with gross average yearly wages, adjusted for the number of effective weekly working hours among women. The labour productivity value estimations included only women considered to constitute the workforce, i.e. those between the ages of 18 and 65.

Due to legal restrictions regarding access to detailed data on prescription medicines, it was not possible to merge this information on a patient level with other direct costs – hence, we report this in a separate category. However, an analysis of the overall costs of prescription medicines produced an estimate of any differences in costs between cases and controls.

Prescription medication in this context include all reimbursed medications, not provided in a hospital setting. For example, anti-nausea medication is often provided in combination with chemotherapy. If that medication is provided in a hospital context, it will be registered in the DNPR. If the same prescription is re-filled at a pharmacy outside the hospital context later, that cost will be registered in this category. However, this category is not limited to cancer-related medications, but all prescription medication collected by the study participants in the study period..

All costs were set to the 2016 price level, and tariffs in both the Danish National Health Service Register for Primary Care and the DNPR were inflated using the combined price and wage index for healthcare services, according to the Danish Regions [20]. Prescription medicine prices were not inflated, as they fluctuate, making price indices difficult to interpret. In the present study, all costs are reported in euros and assume the following exchange rate: EUR 1 = DKK 7.5.

### Statistical analyses

The study followed participants from one year prior to their index date, i.e. date of incident breast cancer diagnosis (1 January 2005 at the earliest), and until 10 years after index date, death, emigration, or end of follow-up (31 December 2016), whichever occurred first.

Average individual direct and indirect costs were calculated on a yearly basis according to index date for cases and controls, respectively. Due to the large number of observations in all categories, a one-sample t-test was applied to determine significance in differences between direct costs among cases and controls.

A secondary analysis estimated the direct costs of metastatic and recurrent breast cancer. Thus, date of metastases or recurrence was defined as the date of breast cancer diagnosis if the patient was diagnosed with metastatic breast cancer (de novo breast cancer), as the date of the 18^th^ treatment of trastuzumab if the patient was identified with more than 17 treatments of trastuzumab, or as the date of re-initiation of treatment with trastuzumab after a six-month break. Direct costs three years after recurrence were estimated. After three years, we do no longer attribute the costs to recurrence but consider it equal to the cost of HER2-positive cancer.

Finally, further analysis included an estimate of the individual average costs of prescription medicines among cases and controls.

Data management and statistical analyses were carried out using SAS statistical software (9.4; SAS Institute, Inc., Cary, NC, USA) on Statistics Denmark’s research computers via a remote server.

## Results

**Fig. 1:**
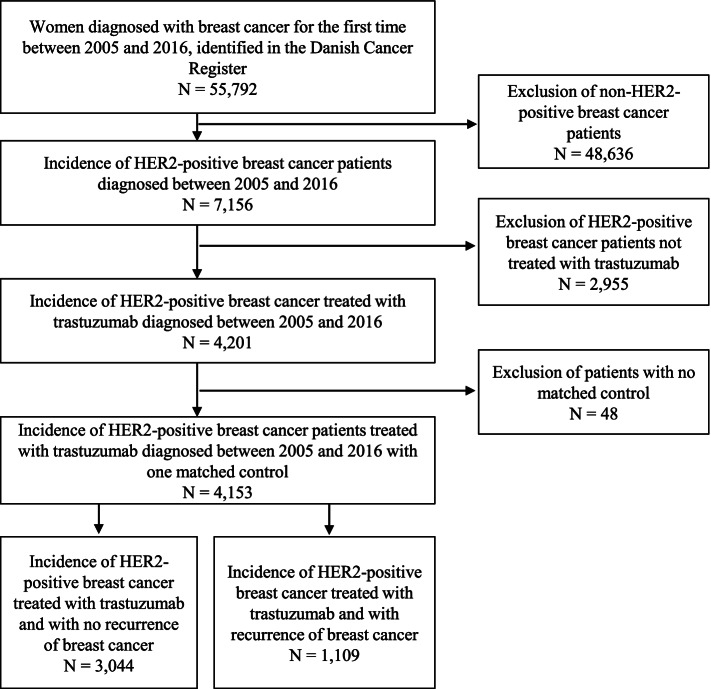
Flowchart of the study population

Between 2005 and 2016, 7,156 incident HER2-positive breast cancer patients were identified in the Danish National Pathology Register. Among those, 4,153 (58%) were treated with trastuzumab within the first year after diagnosis (see flow chart, Fig. 1) and thus were included in the study population. Of these, 1,109 (27%) women were identified as patients with metastatic or recurrent cancer and 3,044 (73%) women were identified as patients without recurrence or metastases.

Basic characteristics of the study population are presented in Table 1. Age and highest obtained education at diagnosis did not vary in correlation to the population with metastatic or recurrent breast cancer and the population without recurrence. However, there was some variation in region of residence. In supplementary materials Table S1, we provide summary statistics of the control pool and matched controls. In Table S2 we present frequency of comorbidities diagnosed two years prior to diagnosis date/index date by ICD-10 chapters.

**Table 1:** Characteristics of the study population

	Total study population	Study population with metastases or recurrence	Study population without metastases or recurrence
	N (%)	N (%)	N (%)
**Total**	4,153 (100%)	1,109 (27%)	3,044 (73%)
**Age at diagnosis**			
Mean (std)	55.8 (11.7)	56.2 (12.5)	55.6 (11.4)
18–40 years	436 (10%)	132 (12%)	304 (10%)
41–50 years	977 (24%)	246 (22%)	731 (24%)
51–60 years	1,242 (30%)	300 (27%)	942 (31%)
61–75 years	1,326 (32%)	362 (33%)	964 (32%)
Over 75 years	172 (4%)	69 (6%)	103 (3%)
**Education**			
Primary or no education	1,106 (27%)	327 (29%)	779 (26%)
Secondary	197 (5%)	53 (5%)	144 (5%)
Short cycle tertiary	1,628 (39%)	411 (37%)	1,217 (40%)
Bachelor’s or equivalent	910 (22%)	246 (22%)	664 (22%)
Master’s or higher	265 (6%)	62 (6%)	203 (7%)
Missing	47 (1%)	10 (0%)	37 (0%)
**Region of residence**			
Capital Region of Denmark	1,180 (28%)	260 (23%)	920 (30%)
Region Zealand	631 (15%)	309 (28%)	322 (11%)
Region of Southern Denmark	945 (23%)	208 (19%)	737 (24%)
Central Denmark Region	985 (24%)	265 (24%)	720 (24%)
North Denmark Region	412 (10%)	67 (6%)	345 (11%)
**TNM classifications**			
**Tumor Size**			
Ta and Tis	-	< 5	7 (0.2%)
T1	1861 (44.8%)	357 (39%)	1,468 (48%)
T2	1426 (34.3%)	348 (38%)	1,117 (37%)
T3	367 (8.8%)	107 (12%)	226 (7.4%)
T4	237 (5.7%)	68 (7.5%)	104 (3.4%)
Tx	208 (5.%)	30 (3.3%)	125 (4.1%)
**Lymph nodes**			
No	1686 (40.6%)	315 (28%)	1,371 (45%)
N1	1424 (34.2%)	395 (36%)	1,113 (37%)
N2	398 (9.6%)	172 (16%)	236 (7.8%)
N3	246 (5.9%)	139 (13%)	132 (4.3%)
Nx	356 (5.9%)	88 (7.9%)	191 (6.3%)
**Metastasis**			
M0	3381 (81.4%)	775 (70%)	2,705 (89%)
M1	216 (5.2%)	199 (18%)	0
Mx	513 (12.4%)	135 (12%)	338 (11%)

Metastasis or recurrence defined as patients with TNM-M code M1 at time of diagnosis, patients who received more than 17 treatments of trastuzumab in the 13 months after diagnosis or patients who received at least six trastuzumab treatments within the first year after diagnosis and at some point afterward discontinued treatment for at least six months, and then initiated trastuzumab treatment again

### Average direct costs

Table 2 presents the annual average direct costs per person among patients with HER2-positive breast cancer treated with trastuzumab compared to matched controls free of breast cancer among the total study population, the population with metastatic or recurrence of breast cancer and the population without metastatic or recurrence of breast cancer.

**Table 2:** Average individual healthcare costs among HER2-positive patients treated with trastuzumab, EUR (CI)

		Year -1	Year 1	Year 2	Year 3	Year 4	Year 5	Year 6	Year 7	Year 8	Year 9	Year 10
Total study population	Case	2,593 (2,445–2,740)	60,555 (59,978–61,132)	19,354 (18,863–19,846)	10,031 (9,456–10,607)	9,097 (8,448–9,746)	8,500 (7,763–9,237)	6,680 (5,983–7,376)	6,575 (5,723–7,427)	6,690 (5,541–7,839)	4,068 (3,221–4,915)	3,961 (2,579–5,343)
	Control	2,228 (1,987–2,469)	2,338 (2,116–2,561)	2,298 (2,042–2,554)	2,275 (2,035–2,514)	2,286 (2,022–2,551)	2,056 (1,799–2,312)	2,101 (1,808–2,394)	2,054 (1,755–2,353)	2,053 (1,621–2,485)	1,701 (1,340–2,062)	1,706 (1,187–2,224)
	*P*-value*	0.01	< .0001	< .0001	< .0001	< .0001	< .0001	< .0001	< .0001	< .0001	< .0001	0.003
	N (cases)	4,153	4,153	3,785	3,212	2,577	2,069	1,611	1,189	775	476	247
Population with metastases or recurrence of breast cancer	Case	2,818 (2,454–3,181)	69,648 (68,235–71,060)	25,475 (24,243–26,707)	18,401 (16,745–20,056)	18,759 (16,754–20,764)	20,385 (17,888–22,881)	17,133 (14,525–19,741)	18,589 (15,205–21,972)	18,420 (14,034–22,806)	11,123 (7,657–14,589)	13,691 (6,227–21,156)
	Control	2,364 (1,741–2,987)	2,548 (2,084–3,013)	2,640 (1,930–3,349)	2,240 (1,785–2,695)	2,108 (1,694–2,522)	1,862 (1,522–2,202)	1,951 (1,404–2,498)	2,077 (1,419–2,736)	1,865 (1,186–2,544)	1,793 (763–2,823)	1,374 (451–2,298)
	*P*-value*	0.22	< .0001	< .0001	< .0001	< .0001	< .0001	< .0001	< .0001	< .0001	< .0001	0.002
	N (cases)	1,109	1,109	1,053	886	684	509	350	240	160	101	42
Population without metastases or recurrence of breast cancer	Case	2,510 (2,359–2,661)	57,171 (56,623–57,718)	16,991 (16,533–17,449)	10,006 (6,497–7,350)	5,759 (5,331–6,187)	4,921 (4,484–5,359)	3,929 (3,505–4,352)	3,478 (3,013–3,943)	3,469 (2,785–4,154)	2,290 (1,860–2,719)	2,116 (1,555–2,676)
	Control	2,178 (1,940–2,416)	2,260 (2,008–2,512)	2,166 (1,940–2,391)	2,287 (2,006–2,569)	2,348 (2,021–2,675)	2,114 (1,795–2,434)	2,140 (1,798–2,482)	2,048 (1,712–2,384)	2,105 (1,587–2,622)	1,678 (1,308–2,048)	1,768 (1,173–2,364)
	*P*-value*	0.0209	< .0001	< .0001	< .0001	< .0001	< .0001	< .0001	< .0001	0.002	0.034	0.403
	N (cases)	3,044	3,044	2,732	2,326	1,893	1,560	1,261	949	615	375	205

^*^T-test for differences in average direct costs between cases and controls

Note: Direct costs included costs of primary care, hospital admissions, outpatient contacts and home care. N controls equal to cases

Metastasis or recurrence defined as patients with TNM-M code M1 at time of diagnosis, patients who received more than 17 treatments of trastuzumab in the 13 months after diagnosis or patients who received at least six trastuzumab treatments within the first year after diagnosis and at some point afterward discontinued treatment for at least six months, and then initiated trastuzumab treatment again

Controls drawn from a pool of breast cancer free danes and matched exact on a 1:1 ratio based on age and education

In the year before diagnosis (year -1), we found no statistically significant difference between metastatic or recurrence cases’ and controls’ average individual direct costs. The differences in total direct costs between cases in the total study population and controls, and between cases without metastatic or recurrence and controls, were statistically significant (*p* = 0.0209); however, the costs were only slightly higher among cases compared to the costs among controls (1.2 times).

In the year of diagnosis, the average direct costs were 25.9 (*p* < 0.001) times higher among cases in the total study population compared to controls, 27.3 (*p* < 0.001) times higher among cases with metastases or recurrence compared to controls and 25.3 (*p* < 0.001) times higher among cases without metastases or recurrence compared to controls, corresponding to differences in actual costs of EUR 58,217, EUR 67,099 and EUR 54,910, respectively.

In subsequent years, the average direct costs remained statistically significantly higher among cases alive compared to controls alive in all study populations. In the 10^th^ year after diagnosis, the average direct costs remained significantly higher among the total study population and among patients with metastases or recurrence. Among all cases, the costs were 2.3 (*p* = 0.003) times higher compared to controls in year 10, whereas the costs were 10.0 (*p* = 0.002) times higher among cases with metastases or recurrence and 1.2 (*p* = 0.402) times higher among cases without metastases or recurrence compared to controls.

### Labour productivity

**Fig. 2:**
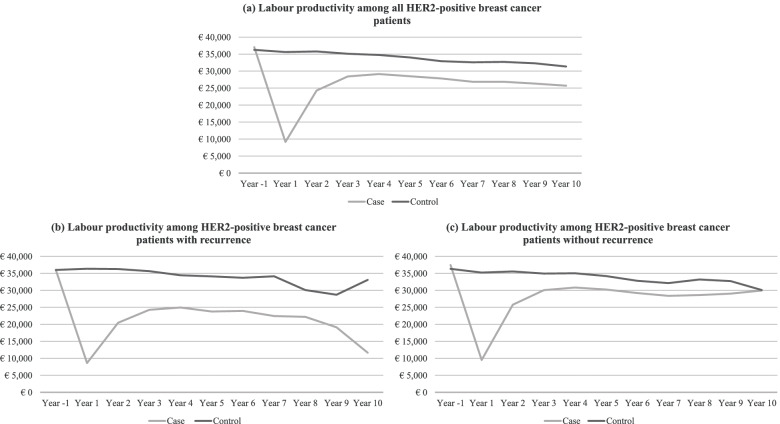
Average individual labour productivity among (**a**) all HER2-positive patients, (**b**) HER2-positive patients with metastases or recurrence and (**c**) HER2-positive patients without metastases or recurrence in the year before diagnosis and 10 years after diagnosis

Figure 2 presents the average individual labour productivity among HER2-positive breast cancer patients compared to controls who were free of breast cancer from year -1 to year 10. In the year prior to index date, cases and controls in all study populations did not differ significantly regarding labour productivity: *p* = 0.180 among the total study population, *p* = 0.99 among cases with metastases or recurrence and *p* = 0.103 among cases without metastases or recurrence. For all three study populations, labour productivity was slightly higher among cases compared to controls.

Among the total study population, labour productivity decreased significantly in the year of diagnosis and remained statistically significantly lower during the entire follow-up period. Among the population of cases with metastases or recurrence, labour productivity was significantly lower throughout the study period; in year 10, labour productivity was 2.8 (*p* < 0.001) times lower among cases compared to controls, whereas cases without metastases or recurrence showed labour productivity comparable to controls from year 9 and onwards (*p* = 0.114).

### Direct costs of recurrence

Among patients experiencing metastases or recurrence, 201 patients (5.5% of cases) were identified with metastatic breast cancer at the time of diagnosis (i.e. de novo breast cancer), while the rest experienced a recurrence after a mean time of 1.5 years. Table 3 presents average individual direct costs three years after recurrence. In the first year after recurrence, the average individual cost increased to EUR 38,383 among cases, compared to EUR 2,129 among their matched controls. Thus, a second spike in direct costs was related to recurrence among breast cancer patients.

**Table 3:** Average individual direct costs after recurrence, EUR (CI)

	Year 1 after recurrence	Year 2 after recurrence	Year 3 after recurrence
Case	38,383 (36,417–40,348)	21,703 (19,929–23,478)	18,486 (16,442–20,530)
Control	2,129 (1,738–2,519)	2,693 (1,837–3,549)	2,350 (1,840–2,860)
*P*-value*	< .0001	< .0001	< .0001
N (cases)	1,096	845	651

^*^T-test for differences in average direct costs between cases and controls. Recurrence defined as patients who received at least six trastuzumab treatments within the first year after diagnosis and at some point afterward discontinued treatment for at least six months, and then initiated trastuzumab treatment again

### Prescription medicine

The average individual costs of prescription medicines are presented in Table 4. In the year prior to index date, the costs of prescription medicines did not vary significantly between cases and controls. In year 1 and year 3, cases had slightly higher costs of prescription medicines compared with controls. In the remaining years, however, we found no differences in costs of prescription medicines between cases and controls. During the entire study period, the average yearly cost of prescription medicine was EUR 343 for cases and EUR 330 for controls.

**Table 4:** Average individual costs of prescription medicines, EUR

		Year -1	Year 1	Year 2	Year 3	Year 4	Year 5	Year 6	Year 7	Year 8	Year 9	Year 10
Total study population	Case	342	401	368	373	364	359	336	324	329	303	277
	Control	346	348	339	324	333	334	328	324	334	310	312
	*P*-value	0.74	0.0004	0.05	0.002	0.09	0.20	0.73	0.996	0.87	0.87	0.52
	N (cases)	4,153	4,153	3,785	3,212	2,577	2,069	1,611	1,189	775	476	247

Controls drawn from a pool of breast cancer free danes and matched exact on a 1:1 ratio based on age and education

## Discussion

Using the Danish national registers, we carried out the first large-scale assessment of the association between HER2-positive breast cancer and direct costs related to metastases or recurrence and no metastases or non-recurrence. Our analyses showed higher direct costs in the 10 years after diagnosis among women with HER2-positive breast cancer who experience metastases or recurrence. During the 10-year follow-up period, patients in the total study population had excess direct costs of EUR 115,000 compared to controls, patients with metastatic or recurrence of breast cancer had excess direct costs of EUR 211,000 compared to controls, and patients without metastases or recurrence had excess direct costs of EUR 89,000 compared to controls. The average individual direct costs were higher in the years after diagnosis among breast cancer patients experiencing metastases or recurrence than among those not experiencing metastases or recurrence. This can partly be explained by the fact that approximately 5% of the study population was diagnosed with de novo breast cancer at incidence.

Due to differences in methodology, study design and included costs – and thereby in the chosen perspective of the analyses – it is somewhat difficult to compare the results of the present study with cost estimates identified in the literature. Bermejo et al. (2020), from Spain, found total costs of HER2-positive/HR-positive to be EUR 290,243 in the five years following diagnosis, while for HER2-positive/HR-Negative it was EUR 249,152. They do not provide a control group to separate the attributable costs, and their cost estimates are based on expert opinion, and not observed costs [10].

A recent study by Sussell et al. [21] investigated the cost of metastatic HER2-positive breast cancer in a US setting. The authors found cumulative direct costs among patients with metastatic HER2-positive breast cancer to be $412,903 (~ EUR 302,000) during the three years after diagnosis, primarily driven by outpatient visits and HER2-targeted therapy drug costs. Although the estimated direct costs were higher than those estimated in this study, the primary drivers were found to be similar, as costs of outpatient contacts including medication dispensed at hospitals constitute 82% of the total costs in this study. The differences in direct costs can partly be explained by the differences in cost of trastuzumab in Denmark and the US. During the study period, the cost of trastuzumab has been 2.5 times higher in the US compared with Denmark (information about US prices has been provided by Roche Denmark). Assuming a similar cost of trastuzumab in Denmark would result in increased outpatient costs corresponding to nearly EUR 200,000 in the three years after diagnosis.

Only patients who received six or more treatments with trastuzumab was included in the patient group. This exclude HER2-positive patients who ceased treatment of trastuzumab before any effect of treatment can be expected [22]. The results are therefore constricted to be the attributable cost of trastuzumab treatment for HER2-positive breast cancer patients.

The strengths of our study include its retrospective register-based design, which included all Danish women diagnosed with HER2-positive breast cancer treated with trastuzumab between 2005 and 2016 with complete information on direct costs up until 2016. By design, the risk of biases related to participation, outcome surveillance or follow-up in our analyses is therefore miniscule. Furthermore, we were able to adjust for age and highest obtained education (and gender by inclusion criteria for pool of controls) by matching the cases and controls on these factors, which limited confounding. While it could have been possible to expand on the matching criteria, it was set to only age and education to avoid selection bias. Direct costs were similar among cases and controls in the year prior to index date, which is essential in studies focused on cost of illness. The number of patients receiving trastuzumab in the study period is consistent with the sales figures of trastuzumab in Denmark, indicating a high validity of the procedure codes in the registers. All in all, the Danish registers are of high completeness, with few missing observations. As there are so few missing observations, we have not concerned ourselves with imputation.

The estimation of indirect costs is based on the average income of women in Denmark, as opposed to the actual reported annual income for each individual. We chose this approach as we have detailed data on which weeks each individual worked. Hence, by using the average income, we can estimate the expected indirect costs which occurred after the cancer diagnosis were given to get a more accurate estimate of the production loss.

However, this study also has a number of limitations that require consideration. Firstly, the phrase “recurrence of breast cancer” is not well defined. The Danish national registers do not include information on recurrence, so we defined “recurrence” by the use of trastuzumab. Therefore, we cannot rule out an element of mis-categorisation of patients as recurrent and non-recurrent, which might have influenced our results. Secondly, the incidence of HER2 breast cancer and the number of patients treated with trastuzumab varied and was particularly lower in the beginning of the study period. This could indicate an irregular use of the SNOMED code for HER2-positive and the procedure code for trastuzumab in the beginning of the study period, although the numbers stabilised in the last part of the study period.

Thirdly, it was not possible to include the costs of prescription medicine in the total direct costs due to data access limitations. However, prescription medicine costs did not seem to vary much between cases and controls during the study period, which indicates that their absence may not have impacted the results significantly.

And lastly, the findings are constrained to the comparison of HER2-positive patients who fit the inclusion criteria and cancer-free control group. Had the control group consisted of a different histologic breast cancer group, the excess costs would likely be lower.

## Conclusion

In conclusion, our study suggests that the direct costs for patients with HER2-positive breast cancer remains significantly higher than the direct costs for controls in the 10 years after diagnosis. Moreover, there is a need for future studies investigating how to identify patients with recurrence, since this information is not defined in the Danish national registers.

## Supplementary Information

**Additional file 1: Table S1.** Summary statistics of the matched and unmatched control population. Differences between group tested with t-test or chi^2^.

**Additional file 2: Table S2.** Summary statistics of diagnosis of the study participants given as primary diagnosis in hospital setting in the two years prior to diagnosis date/index date.

## Data Availability

The data that support the findings of this study are available from Statistics Denmark’s Research Service. However, restrictions apply to the availability of these data, which were used under license/authorisation for the current study and so are not publicly available. Additional data analyses are available from the authors upon reasonable request and with permission of Statistics Denmark’s Research Service.

## References

[1] Ferlay J, Colombet M, Soerjomataram I, Dyba T, Randi G, Bettio M (2018). Cancer incidence and mortality patterns in Europe: Estimates for 40 countries and 25 major cancers in 2018. Eur J Cancer.

[2] World Health Organization (2020). Denmark, number of new cancer cases in 2018.

[3] Nordcan. Cancer stat fact sheets, Nordic countries - Breast cancer 2016. https://www-dep.iarc.fr/NORDCAN/english/frame.asp (Accessed 21 Apr 2021).

[4] World Health Organization (2020). World, number of new cancer cases in 2018.

[5] Beau A-B, Andersen PK, Vejborg I, Lynge E (2018). Limitations in the Effect of Screening on Breast Cancer Mortality. J Clin Oncol.

[6] Kohler BA, Sherman RL, Howlader N, Jemal A, Ryerson AB, Henry KA, et al. Annual Report to the Nation on the Status of Cancer, 1975–2011, Featuring Incidence of Breast Cancer Subtypes by Race/Ethnicity, Poverty, and State. J Natl Cancer Inst 2015;107. 10.1093/jnci/djv048. 10.1093/jnci/djv048 PMC4603551 25825511

[7] Patel A, Unni N, Peng Y. The Changing Paradigm for the Treatment of HER2-Positive Breast Cancer. Cancers (Basel) 2020;12. 10.3390/cancers12082081. 10.3390/cancers12082081 PMC7464074 32731409

[8] Dumas A, Vaz Luis I, Bovagnet T, El Mouhebb M, Di Meglio A, Pinto S (2020). Impact of Breast Cancer Treatment on Employment: Results of a Multicenter Prospective Cohort Study (CANTO). JCO.

[9] Tartari F, Santoni M, Pistelli M, Berardi R (2017). Healthcare cost of HER2-positive and negative breast tumors in the United States (2012–2035). Cancer Treat Rev.

[10] Bermejo de las Heras B, Cortes Ramon y Cajal J, GalveCalvo E, de la Haba Rodriguez J, Garcia Mata J, Moreno Anton F (2020). The economic burden of metastatic breast cancer in Spain. Eur J Hosp Pharm.

[11] Pedersen CB (2011). The Danish Civil Registration System. Scand J Public Health.

[12] Lynge E, Sandegaard JL, Rebolj M (2011). The Danish National Patient Register. Scand J Public Health.

[13] Gjerstorff ML (2011). The Danish Cancer Registry. Scand J Public Health.

[14] Erichsen R, Lash TL, Hamilton-Dutoit SJ, Bjerregaard B, Vyberg M, Pedersen L (2010). Existing data sources for clinical epidemiology: the Danish National Pathology Registry and Data Bank. Clin Epidemiol.

[15] Andersen JS, Olivarius NDF, Krasnik A (2011). The Danish National Health Service Register. Scand J Public Health.

[16] Jensen VM, Rasmussen AW (2011). Danish education registers. Scand J Public Health.

[17] Jensen JD, Knoop A, Ewertz M, Laenkholm A-V (2012). ER, HER2, and TOP2A expression in primary tumor, synchronous axillary nodes, and asynchronous metastases in breast cancer. Breast Cancer Res Treat.

[18] Systemisk behandling af brystkræft III – palliativ og systemisk behandling af metastaserende brystkræft (MBC) [Systemic treatment of Breast Cancer – III – palliative and systemic treatment of metastatic breast cancer (MBC)] 2021.

[19] Systemisk behandling af brystkræft - II – (neo)adjuverende systemisk behandling af tidlig brystkræft [Systemic treatment of Breast Cancer II – (neo)adjuvant systemic treatment of early-stage breast cancer] 2021.

[20] Danish Regions. Økonomisk Vejledning 2017 n.d. http://www.regioner.dk/aftaler-og-oekonomi/oekonomisk-vejledning/oekonomisk-vejledning-2017 (Accessed 7 Oct 2020).

[21] Sussell JA, Sheinson D, Wu N, Shah-Manek B, Seetasith A (2020). HER2-Positive Metastatic Breast Cancer: A Retrospective Cohort Study of Healthcare Costs in the Targeted-Therapy Age. Adv Ther.

[22] Chen L, Zhou W, Hu X, Yi M, Ye C, Yao G (2019). Short-duration versus 1-year adjuvant trastuzumab in early HER2 positive breast cancer: A meta-analysis of randomized controlled trials. Cancer Treat Rev.

