# Supplementary material for: Cost of illness of HER2-positive and metastatic and recurrent HER2-positive breast cancer – a Danish register-based study from 2005 to 2016
Source: BMC Health Serv Res. 2022 Jun 4;22:745. doi: 10.1186/s12913-022-08143-7 (PMC9167500; doi:10.1186/s12913-022-08143-7)
Supplement: Supplementary file 1 — Additional file 1: Table S1. Summary statistics of the matched and unmatched control population. Differences between group tested with t-test or chi2. [file 12913_2022_8143_MOESM1_ESM.docx]

Table S1: Summary statistics of the matched and unmatched control population. Differences between group tested with t-test or chi^2^.

|  | Not matched controls | Matched controls, | p-value*^2^* |
| --- | --- | --- | --- |
|  | N (%) | N (%) |  |
| **Total** | 87,812 (100%) | 4,152 (%) |  |
| **Age at observation** |  |  | <0.001 |
| Mean (sd) | 62.56 (13.39) | 55,2 (11.7) |  |
| 18 - 40 Years | 3,420 (3.9%) | 403 (9.7%) |  |
| 41 - 50 Years | 12,016 (14%) | 941 (23%) |  |
| 51 - 60 Years | 20,826 (24%) | 1,267 (31%) |  |
| 61 - 75 Years | 33,850 (39%) | 1,349 (32%) |  |
| Over 75 Years | 17,700 (20%) | 192 (4.6%) |  |
| **Education** |  |  | <0.001 |
| Primary or no education | 32,015 (39%) | 1,106 (27%) |  |
| Secondary | 2,251 (2.7%) | 197 (4.8%) |  |
| Short cycle tertiary | 29,864 (36%) | 1,628 (40%) |  |
| Bachelor of equivalent | 14,389 (18%) | 910 (22%) |  |
| Master or higher | 3,635 (4.4%) | 265 (6.5%) |  |
| Missing | 5658 (6.4%) | 46 (1,1%) |  |
| **Region of residence** |  |  | 0.2 |
| Capital Region of Denmark | 25,726 (30%) | 1,322 (32%) |  |
| Central Denmark Region | 18,942 (22%) | 899 (22%) |  |
| North Denmark Region | 9,231 (11%) | 429 (10%) |  |
| Region of Southern Denmark | 18,353 (21%) | 872 (21%) |  |
| Region Zealand | 13,482 (16%) | 629 (15%) |  |
| Missing | 2078 (2.3%) | - |  |

Note: Age of matched controls differ with cases because age is measure by year from birth, while age presented in Table 1 is age at diagnosis.
