# Supplementary material for: Cost of illness of HER2-positive and metastatic and recurrent HER2-positive breast cancer – a Danish register-based study from 2005 to 2016
Source: BMC Health Serv Res. 2022 Jun 4;22:745. doi: 10.1186/s12913-022-08143-7 (PMC9167500; doi:10.1186/s12913-022-08143-7)
Supplement: Supplementary file 2 — Additional file 2: Table S2. Summary statistics of diagnosis of the study participants given as primary diagnosis in hospital setting in the two years prior to diagnosis date/index date. [file 12913_2022_8143_MOESM2_ESM.docx]

Table S2: Summary statistics of diagnosis of the study participants given as primary diagnosis in hospital setting in the two years prior to diagnosis date/index date

Note: Chapter DZ was excluded due to the general nature of codes in this chapter.

| ICD-10 chapter | Description | No metastases or recurrence | | Metastases or recurrence | | Matched Controls | |
| --- | --- | --- | --- | --- | --- | --- | --- |
|  |  | n | Ratio | n | Ratio | n | Ratio |
| Not provided | - | 20 | 0.66 | <5 | - | 10 | 0.24 |
| DA | Infectious disease | 22 | 0.72 | 7 | 0.63 | 40 | 0.96 |
| DB | Parasitic disease | 7 | 0.23 | <5 | - | 9 | 0.22 |
| DD | Neoplasma | 169 | 5.55 | 73 | 6.58 | 144 | 3.47 |
| DE | Metabolic disease | 103 | 3.38 | 42 | 3.79 | 148 | 3.56 |
| DF | Psycological disorder | 14 | 0.46 | 6 | 0.54 | 23 | 0.55 |
| DG | Neurologic disease | 89 | 2.92 | 43 | 3.88 | 122 | 2.94 |
| DH | Eye disease | 185 | 6.08 | 61 | 5.50 | 217 | 5.23 |
| DI | Cardivascular disease | 169 | 5.55 | 64 | 5.77 | 275 | 6.62 |
| DJ | Respatory disease | 72 | 2.37 | 26 | 2.34 | 130 | 3.13 |
